# Dumbbell Spinal Desmoid Tumor Mimicking a Giant Schwannoma: Case Report and Literature Review

**DOI:** 10.3390/jcm14217596

**Published:** 2025-10-26

**Authors:** Hajar Nafidi, Rossella Rispoli, Stefano Pizzolitto, Corrado Iaccarino, Giacomo Pavesi, Barbara Cappelletto

**Affiliations:** 1 Division of Neurosurgery, Department of Biomedical, Metabolic and Neural Sciences, University of Modena and Reggio Emilia, University Hospital of Modena, 41125 Modena, Italy; hajar.nafidi@gmail.com (H.N.);; 2 Spine and Spinal Cord Surgery Unit, Department of Head, Neck, and Neurological Sciences, University Hospital of Udine, 33100 Udine, Italy; barbara.cappelletto@asufc.sanita.fvg.it; 3 Department of Pathology, University Hospital of Udine, 33100 Udine, Italy; stefano.pizzolitto@asufc.sanita.fvg.it

**Keywords:** desmoid tumor, spinal fibromatosis, dumbbell tumor, thoracic tumor, β-catenin, surgical resection, adjuvant therapy, recurrence

## Abstract

**Background/Objectives:** Desmoid tumors (DTs) are rare benign soft tissue neoplasms characterized by local aggressiveness and high rate of recurrence. Spinal localization is only anecdotally reported in the literature. When presenting in a dumbbell-shaped configuration, they can mimic neurogenic tumors. **Methods:** We report a rare case of a spinal intracanalar–intrathoracic DT, initially suspected to be a schwannoma, and review the literature. **Results:** A 24-year-old asymptomatic man was incidentally found to have a mediastinal mass on chest X-ray. CT and MRI revealed a left thoracic paravertebral mass (T9–T12), with intracanalar extension through the T10–T11 foramen, suggestive of a dumbbell-shaped neurogenic tumor. After embolization, the patient underwent surgery via a posterior combined intracanalar and endothoracic approach. Histology and immunohistochemistry analysis identified the tumor as a desmoid type fibromatosis (β catenin positive; S100, CD34, SMA negative). Follow-up MRI at 8, 12, and 18 months showed stable residual intrathoracic mass. **Conclusions:** To date, only 36 cases of spinal DTs have been reported in the literature, of which only 6 exhibited dumbbell morphology. Immunohistochemical and molecular pathological testing is essential for diagnosis. Although wide resection is preferred, anatomical limitations often necessitate marginal or subtotal surgery, which increases the risk of recurrence (24–77%). Our review showed a 29% overall recurrence rate (50% after subtotal, 29% marginal, 20% wide resection). Adjuvant radiotherapy or systemic therapies may help improve outcomes. Spinal DTs pose significant diagnostic and therapeutic challenges. In the absence of established guidelines, management should be individualized and multidisciplinary. Lifelong follow-up is essential due to the high risk of recurrence.

## 1. Introduction

Desmoid tumor (DT), also known as desmoid fibromatosis or aggressive fibromatosis, is defined by the World Health Organization (WHO) as a rare type of soft-tissue/fibroblastic tumor [1] that accounts for less than 3% of all soft tissue tumors and about 0.03% of all neoplasms [2,3,4], affecting 2–4 people per million per year. The incidence of DTs is significantly higher in females compared to male patients [1,2,3,4,5,6,7,8,9,10,11,12,13,14], with female cases being 2.2–3.9 times the number of male cases [15]. A dumbbell-shaped tumor is a type of neoplasm that spans both the inner and outer spinal canal [16].

DTs are typically non-metastasizing, relatively slow growing, classified as intermediate tumors with benign systemic effects but locally invasive histology and high recurrence rates [1,17]. Clinically, DTs often present as a progressively expanding soft tissue mass causing discomfort or pain due to compression of adjacent vascular, nervous, or muscular structures [18,19]. The etiology of DTs is multifactorial, including genetic, endocrine and physical factors. Surgical trauma may accelerate the development of DTs, though the precise pathogenesis of which remains unknown [9]. Two principal types of desmoid fibromatosis have been identified: the sporadic type, which is the most common, and the genetic type, which is usually associated with Gardner syndrome [20].

The diagnosis and evaluation of the tumor are achieved through magnetic resonance imaging (MRI), while treatment requires a multidisciplinary approach [3,4]. Due to the rarity of DTs, no standardized management guidelines are currently available. The primary treatment consists of wide surgical excision of the tumor. Multimodal therapeutic approaches, such as radiation therapy, hormonal therapy, or cytotoxic chemotherapy, are increasingly employed as primary or as adjuvant therapies in cases of incomplete resection or recurrence [21]. Additionally, there is a growing emphasis on more conservative and individualized management strategies.

DTs arise from musculoaponeurotic structures and can develop in any part of the body [10]. In neurosurgical literature, DTs are only anecdotally described in sporadic case reports [5,6,7,8,9,10,11,22,23,24,25,26,27,28,29,30,31,32,33,34,35,36,37,38,39,40,41,42,43,44,45,46,47]. Spinal desmoid fibromatosis represents a unique subset of fibromatosis with a paucity of clinical data that limits a comprehensive understanding of its presentation and treatment.

Distinguishing these tumors from other lesions can be challenging based on radiological or even histological features. Therefore, adequate tissue sampling and immunohistochemical studies are essential for an accurate diagnosis.

We present a case of a sporadic spinal intracanalar-intrathoracic dumbbell desmoid tumor mimicking a giant schwannoma, alongside a comprehensive review of all previously reported cases to date [5,6,7,8,9,10,22,23,24,25,26,27,28,29,30,31,32,33,34,35,36,37,38,39,40,41,42,43,44,45,46,47].

## 2. Case Report

A 24-year-old man underwent a medical examination required by the occupational doctor due to the onset of contact-induced allergic dermatitis from flour and a routine chest X-ray documented, as an incidental finding, a left posterior retrocardiac mass

A further thoracic contrast enhanced computerized tomography (CT) scan was performed and demonstrated a 74 × 67 × 92 mm left paravertebral mass, expanding from T9 to T12, hypodense, with slight post-contrast enhancement, extending into the spinal canal through the ipsilateral neural foramen T10–T11, without evidence of bone erosion. No significant changes in lung parenchyma, and no mediastinal lymphadenopathy were observed.

**Figure 1:**
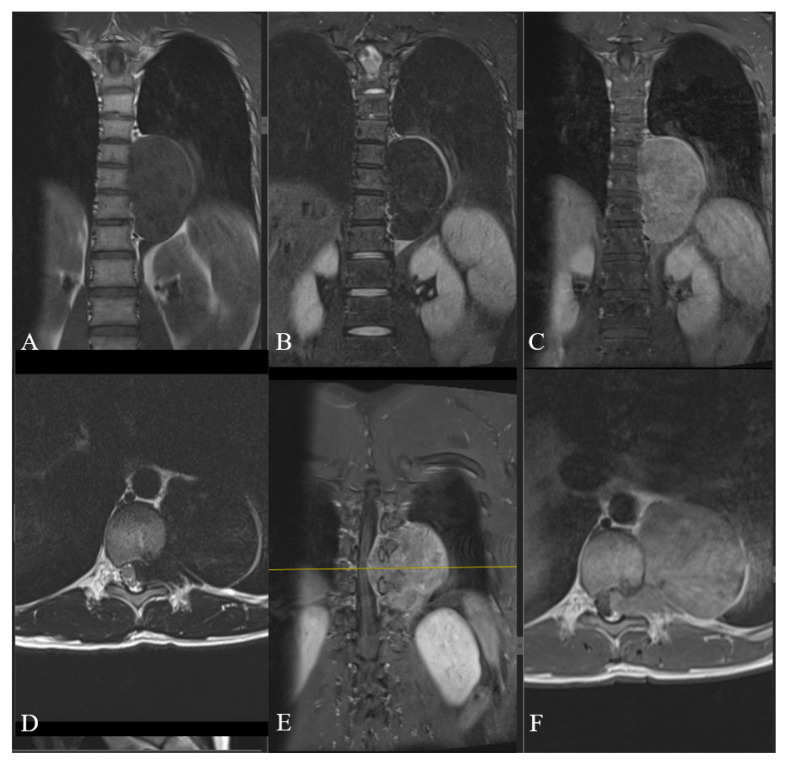
MRI showing an approximately 61 × 56 × 93 mm left paravertebral solid mass with a non-homogeneous iso- to hyperintense signal on T1-weighted images (**A**), hypointense signal on T2-weighted images (**B**), and homogeneous post-contrast enhancement (**C**). The mass extends through the T10–T11 neural foramen, displacing the spinal cord to the right, which maintains regular signal intensity (**D**). The intrathoracic portion anteriorly and laterally compresses the mediastinum, pleura, and lung parenchyma, with a cleavage plane visible on T1-weighted post-contrast coronal (**E**) and axial (**F**) sections. The yellow line indicates the corresponding axial section (**F**), where the mass reaches its maximal extension.

The MRI of the thoracic spine confirmed the presence of an approximately 61 × 56 × 93 mm left paravertebral solid mass, extended through the T10–T11 neural foramen, causing significant spinal canal involvement. The lesion markedly imprinted and displaced to the right side the spinal cord that however maintained regular signal intensity. The neoformation presented well-defined margins, exhibited T2 hypointense and T1 non-homogeneous iso-hyperintense signal, and homogeneous post-contrast enhancement. The intrathoracic portion anteriorly and laterally imprinted the mediastinum, pleura and lung parenchyma with a distinct cleavage plane visible. Additionally, a subtle hyperintensity in STIR sequences at the left pedicle and transverse process of T10 was observed. The lesion was considered a dumbbell-shaped neurogenic tumor (Figure 1).

Therefore, the patient was referred to our center for evaluation. He reported no history of trauma, previous surgery, significant family history, or syndromic features suggestive of Gardner syndrome. His neurological exam, including motor, sensory and reflex testing was normal.

**Figure 2:**
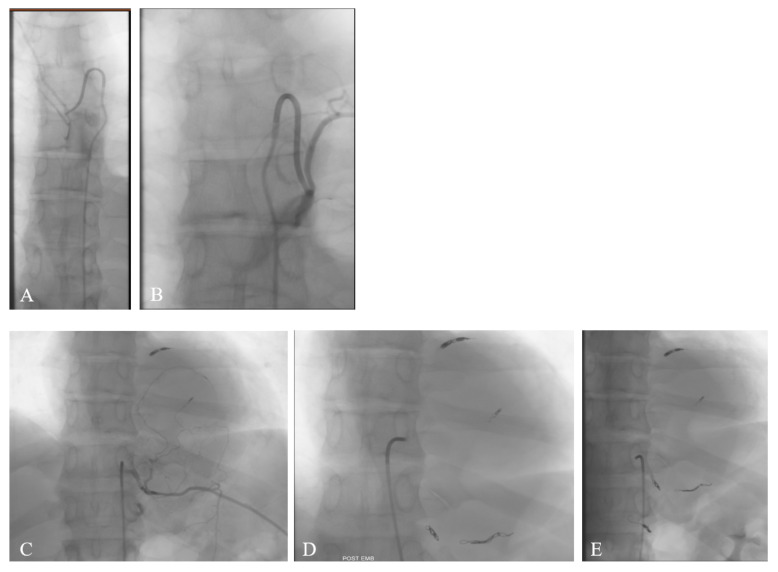
Angiography demonstrated that the arterial supply to the anterior spinal artery originated from the left T7 and T9 (**A**,**B**), and the right T12 and L3. Embolization of the tumor’s feeding branches from the left T10, T11, and T12 was performed using multiple coils (**C**–**E**).

We requested the angiographic study to assess the origin of the anterior spinal artery (Figure 2A,B), as this information crucial for surgical planning and minimizing the risk of spinal cord ischemic damage. The angiography demonstrated that the vascular afferences to the anterior spinal artery originated from the left T7 and T9, and the right T12 and L3 arteries. Based on the vascular pattern and tumor hypervascularization demonstrated during the angiographic study, the interventional radiologist decided to proceed with embolization (Figure 2C–E) to reduce intraoperative bleeding and facilitate tumor resection. Embolization of the tumor’s feeding branches from the left T10, T11, and T12—avoiding the left T9 supply to the anterior spinal artery—was performed using multiple coils, successfully excluding the blood supply to the mass.

The patient underwent surgery, as planned, via a posterior combined intracanalar and endothoracic approach, using intraoperative neurophysiological monitoring (IONM). A left T10 laminectomy was performed, revealing an intracanalar, grayish, non-bleeding, elastic, ligneous, firm, and well-encapsulated mass, tapered by the T10 nerve root. The mass was carefully resected, preserving the nerve root. Subsequently, through a small thoracotomy between the left 10th and 11th ribs, the voluminous endothoracic portion, exhibiting the same appearance of the intracanalar mass, was dissected and removed with difficulty due to firm attachments to the surrounding tissues. Intraoperatively it was evident that the tumor did not originate from the T10 nerve root and had no adhesion to the spinal canal structures but was instead firmly anchored to the paravertebral soft tissues. At the end of surgery, the T10 nerve root appeared deformed and tortuous, but preserved, and the left lung was intact. A mild reduction in MEP amplitude was documented by IONM. An incisional biopsy, performed at the beginning of the procedure, showed rare spindle cells, suggestive of a spindle cell neoplasm.

**Figure 3:**
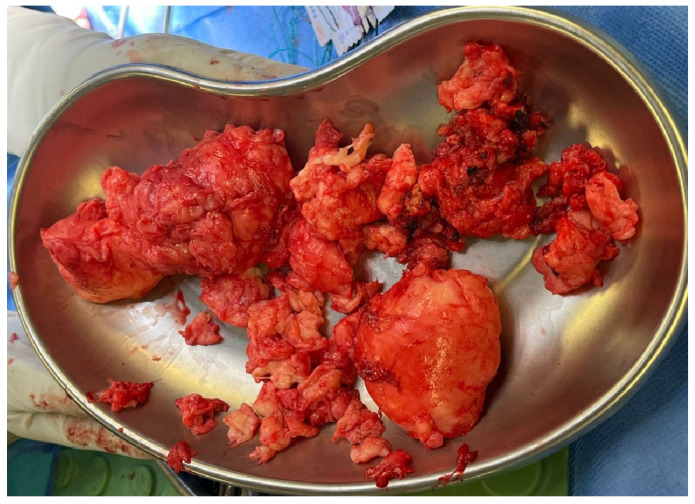
Post-resection photo showing multiple irregular, lobulated pieces of excised tissue in a kidney-shaped surgical tray. The specimen was sent for histological analysis.

The excised mass (Figure 3) was sent to pathology for histological analysis.

**Figure 4:**
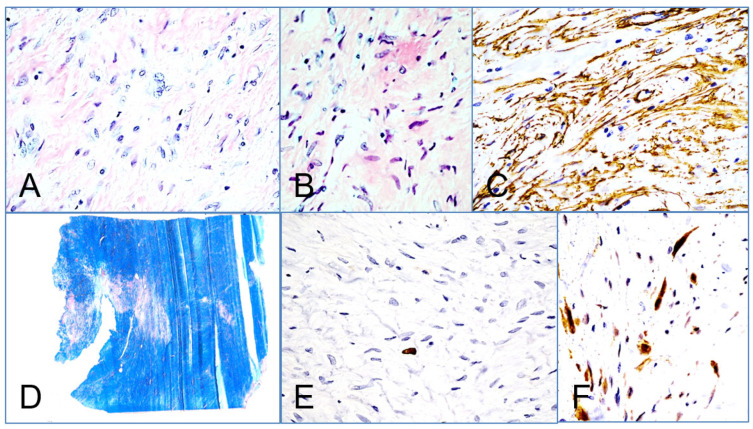
Histological and immunohistochemical analysis led to the diagnosis of desmoid-type fibromatosis. (**A**): The tumor shows low to moderate cellularity consisting of elongated, spindle-shaped fibroblast-like cells embedded in an abundant collagenous desmoid stroma (Hematoxylin and Eosin, 400×). (**B**): The nuclei display no significant pleomorphism or atypia; necrosis, mitotic activity, and hypervascularity are absent (Hematoxylin and Eosin, 400×). (**C**): Immunohistochemical staining demonstrates focal positivity for smooth muscle actin (SMA, 200×). (**D**): Gomori trichrome staining highlights strong and diffuse positivity of the collagenous desmoid stroma (40×). (**E**): Ki-67 immunostaining reveals a proliferative index of less than 1% (400×). (**F**): Nuclear immunoreactivity for β-catenin, characteristic of fibromatoses, is observed (400×).

The tumor had the phenotypic staining and immunohistochemical characteristics of a desmoid type fibromatosis. Histologically, the tumor revealed low to moderate cellularity, composed of elongated, spindle-shaped fibroblast-like cells embedded within a collagen-rich desmoid stroma. The nuclei appeared bland, without significant pleomorphism or atypia, and there was no evidence of necrosis, mitotic activity, or hypervascularity. Immunohistochemically, focal positivity for smooth muscle actin (SMA) was observed, while staining for S-100 protein, STAT6, CD34, and desmin was negative, effectively excluding differential diagnoses such as neurofibroma, leiomyoma, primary endothelial tumors, solitary fibrous tumors, and metastases. Gomori trichrome staining demonstrated strong and diffuse positivity within the collagenous stroma, supporting the fibrous nature of the lesion. The Ki-67 proliferative index was below 1%, indicating a very low rate of cellular proliferation. Additionally, nuclear immunoreactivity for β-catenin, a hallmark of fibromatoses, was clearly detected, further substantiating the diagnosis. All relevant histopathological and immunohistochemical findings are depicted in Figure 4.

**Figure 5:**
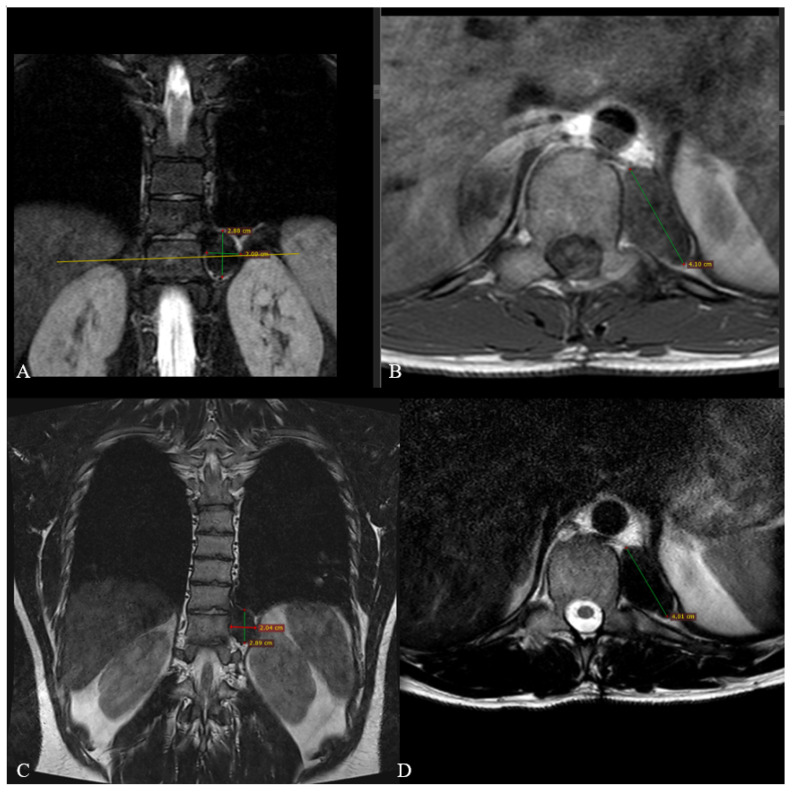
The post-operative MRI at 8 months showed a residual 20.1 × 28.8 × 41.0 mm lobule near the T11 vertebral body (**A**,**B**); the yellow line indicates the corresponding axial section (**B**). The MRI at 18 months demonstrated stable dimensions of 20.4 × 28.9 × 41.1 mm (**C**,**D**).

The patient had an uneventful postoperative recovery and was discharged on postoperative day 7. The close follow-up imaging was advised to monitor for recurrence. At 8 months, MRI revealed a small residual/recurrent lobule (20.1 × 28.8 × 41.0 mm) near the left lateral wall of the T12 vertebral body (Figure 5A,B). The patient was referred to a specialized oncological center for evaluation, comprehensive management, and consideration of further treatment if indicated. However, despite our recommendations, the patient declined referral and opted for clinical and radiological follow-up instead. The lesion remained stable at 12 months (20.9 × 30.1 × 40.4 mm) and at 18 months (20.4 × 28.9 × 41.1 mm) follow-up (Figure 5C,D). The slight variation in measurements was attributed to the irregular shape of the lesion.

**Figure 6:**
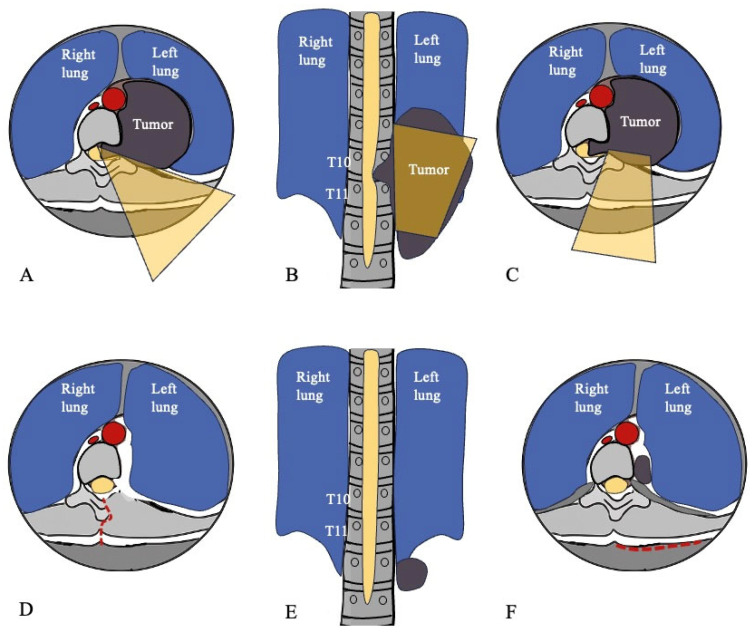
MRI-corresponding sketch for preoperative planning and postoperative outcome. (**A**) First phase: removal of the intracanalar component. The yellow triangle indicates the expected surgical field vision of the spinal cord and nerve root after left T10 laminectomy. (**B**,**C**) Second phase: removal of the intrathoracic component. The yellow trapezoid indicates the expected surgical field vision of the mass after a small thoracotomy between the left 10th and 11th ribs. (**D**) Left T10 laminectomy and complete removal of the intracanalar component; the red dotted line corresponds to the first incision. (**E**,**F**) Residual intrathoracic component at the T12 level; the red dotted line corresponds to the second incision.

The final figure provides a schematic illustration of the surgical strategy (Figure 6A–C) and the residual/recurrent lesion (Figure 6D–F), summarizing the approach and the surgical outcome while highlighting how the procedure targeted the lesion while preserving surrounding structures.

## 3. Discussion

This report describes a desmoid tumor presenting as a spinal intracanalar-endothoracic dumbbell tumor, mimicking a giant schwannoma. We also provide a literature review of previously reported cases: 32 studies have been published up to date [5,6,7,8,9,10,22,23,24,25,26,27,28,29,30,31,32,33,34,35,36,37,38,39,40,41,42,43,44,45,46,47]. The clinical data from all documented cases of spinal desmoid fibromatosis are summarized in Table 1. The clinical characteristics and outcomes observed in our patient are consistent with those described in prior cases of this tumor type.

### 3.1. Diagnosis

#### 3.1.1. Definition, Historical Background, and Epidemiology

Desmoid tumors (DTs), also known as desmoid-type fibromatosis, are rare fibroblastic neoplasms of intermediate biological potential, characterized by aggressive local behavior with and high recurrence rate but without metastatic potential [1,17]. MacFarlane first described a desmoid tumor in 1832 and 6 years later Müller coined the term desmoid, meaning “tendon-like”, to emphasize the tumor’s firm consistency [12]. However, the first reported case of a paraspinal desmoid tumor did not appear until 1961 [22].

DTs are rare: their incidence is low (2–4/million/year), accounting for less than 3% of all soft tissue tumors and approximately 0.03% of all neoplasms [2,3,4]. The incidence of DTs varies by age and gender. In our data analysis, patients’ ages ranged from 19 months to 71 years, with a mean age of 39 years. Twenty-five patients (69%) were females and 11 (31%) were males (F:M, 6:1). Reitamo et al. reported that 80% of desmoid fibromatosis occurs in females, with 50% of these patients between 30 and 50 years old, suggesting that these tumors may respond to certain hormonal profiles [48]. Bektas et al. in their comprehensive review also reported a female predominance, with female cases being 2.2–3.9 times the number of male cases [15]. In the pediatric population (age < 18 years), most DTs are extra-abdominal and occur equally in males and female [13]. To date, only 7 (19%) pediatric patients with spinal DT have been reported.

#### 3.1.2. Classification

The World Health Organization (WHO) classifies desmoid tumors (DTs) as an intermediate, locally aggressive, rare type of soft-tissue tumor [1], arising from the muscular-aponeurotic structures in any part of the body. They can be classified as extra-abdominal (trunk and limbs), located on the abdominal wall, or intra-abdominal [10]. The most common locations of DTs include the extremities, trunk musculature, head and neck, and the abdominal cavity [11,48,49]. In the neurosurgical literature, DTs are only anecdotally described: those with spinal involvement are exceptionally rare, with only 36 reported cases in the literature [5,6,7,8,9,10,22,23,24,25,26,27,28,29,30,31,32,33,34,35,36,37,38,39,40,41,42,43,44,45,46,47].

#### 3.1.3. Review of Reported Spinal Cases

To the best of our knowledge, among the 36 reported cases, 18 (50%) involved postoperative paraspinal fibromatosis resulting from surgical trauma: 11 of these cases included spinal fixation hardware, while 7 cases were operated for other tumor resections (meningioma, schwannoma, hemangioma, and ependymoma). In Table 1, gray-highlighted rows indicate post-surgical cases. The remaining 18 patients had no history of surgical trauma associated with DTs: 16 (44%) had de novo tumors, and 2 (6%) patients were diagnosed with Gardner syndrome.

The tumor was most commonly located in the cervical and thoracic spine, with 13 in the cervical spine, 11 in the thoracic spine, 5 at the cervicothoracic junction, 4 in the lumbar spine, 2 at the thoracolumbar junction, and 1 at the occipito-cervical junction. Additionally, one thoracic mass was reported as intramedullary, and one cervical tumor originated from the vertebral body. Paraspinal involvement was observed in 28 cases. Even paraspinal DTs without spinal extensions may require neurosurgical interventions to preserve spinal stability. For example, Hood et al. reported a case where an occipito-cervical instrumented fusion was performed after the extensive resection of a cervical paraspinal recurrent DT, to maintain spinal stability and prevent kyphosis [40].

Only 6 patients presented with both paraspinal and intraspinal tumor extension, presenting as dumbbell-shaped tumors. A dumbbell-shaped tumor refers to a neoplasm that spans both the inner and outer spinal canal [16]. Most are neurogenic tumors and are characterized by a dumbbell or hourglass configuration with encroachment on anatomical structures such as the intervertebral foramen or dura. Nerve-sheath tumors are the most common type of dumbbell-shaped tumor in adults, accounting for about 80% [50]. The other non-neurogenic dumbbell tumors include angiomas, meningiomas, chondrosarcoma, and other rare entities like desmoid fibromatosis [50].

The present case represents a rare form of desmoid tumor that fits the neurosurgical definition of a dumbbell-shaped tumor, featuring an intraspinal portion and a large intrathoracic extension.

#### 3.1.4. Pathogenesis and Molecular Mechanisms

DTs are also known as desmoid fibromatosis or aggressive fibromatosis. The term “aggressive” is often preferred because better reflects the tumor biology with its locally invasive and morbid behavior [51].

The pathological mechanisms underlying the development of DT remain unclear. Two principal types of DTs have been identified: (1) the sporadic type, which is most common, and its pathogenesis is associated with mutations in the β-catenin gene (CTNNB1) [52]; (2) the genetic type, which is more frequently observed in Gardner syndrome [20], and linked to the adenomatous polyposis coli (APC) gene mutation [22].

β-catenin is a protein known to play a major role in bone healing following injury. [53] Individuals found to have a sporadic desmoid tumor often report a remote history of trauma near the tumor site, suggesting a potential link between β-catenin and desmoid tumor pathogenesis [5]. Surgical trauma may also accelerate the development of DTs at the operation site, although the underlying mechanisms remain unknown [9]. Indeed, a key diagnostic characteristic of desmoid tumors is the presence of somatic β-catenin or APC gene mutations, resulting in intranuclear accumulation of β-catenin [19,52], leading to a downstream activation of Wnt signaling pathways and subsequent effect on target genes transcription, such as c-myc, cyclin D1 and cox-2, promoting uncontrolled fiber cell proliferation and differentiation [52,54]. A history of prior surgery or trauma at the tumor site is frequently reported, supporting the hypothesis that aberrant wound-healing pathways and mechanical stress may serve as initiating or promoting events [5,9,53].

#### 3.1.5. Clinical Presentation

DTs typically present as a progressively expanding soft tissue mass that is uncomfortable or painful due to compression of adjacent vascular, nervous, or muscular structures [18,19]. The clinical presentation varies depending on the location of the neoplasm. Patients rarely present with a painful or painless limp, pain, or neurological symptoms [13]. From our data analysis, among the 36 published cases of spinal fibromatosis, 24 (67%) presented with a painful limp, 8 (22%) with a painless limp, 8 (22%) had pain not associated with a limp, 7 (19%) had neurological deficits (sensory/motor or paresthesia), and 2 patients (6%) were asymptomatic. Overall, the tumor’s slow growth and tendency to invade contiguous structures tends to reduce morbidity, unless it encroaches on vital structures [55,56].

Our patient reported no symptoms, likely due to the silent and slow growth of the neoplasm into the thorax.

#### 3.1.6. Radiological Features and Differential Diagnosis

MRI is the radiological gold standard for diagnosis, evaluation of disease extent, detection of recurrence, and longitudinal follow-up [57,58]. Typical findings include low-to-intermediate signal intensity on T1-weighted sequences (similar to muscle), heterogeneously high signal intensity on T2-weighted sequences (slightly higher than muscle), and moderate to intense post-contrast enhancement, characteristic of fibrotic and collagenous tumors [57,58,59]. However, these features are nonspecific and may mimic other entities, such as fibrosarcoma, solitary fibrous tumor, lymphoma, granulomatous inflammation, desmoplastic fibroma of the bone, myxoma [19]. Furthermore, the differential diagnosis, especially of a dumbbell-shaped desmoid tumor, includes schwannoma, fibrous meningioma with extensive collagenous transformation, nodular fasciitis, and metastasis [24,50,60,61,62]. CT imaging is useful for assessing bone involvement, although DTs typically spare osseous structures.

Certain subtle features may raise suspicion for a desmoid tumor rather than a dumbbell schwannoma, including the lack of true neural foraminal widening or bony remodeling, which are commonly seen in schwannomas, and the presence of a homogeneous or collagenous stroma signal on MRI, in contrast to the usually more cystic or heterogeneous appearance of schwannomas. However, distinguishing desmoid tumors from other lesions based solely on radiological appearance can be exceedingly difficult, and imaging should be interpreted alongside a thorough review of the patient’s medical history, adequate tissue sampling, pathological findings and immunohistochemical studies, to ensure an accurate diagnosis.

In this case, the lesion presented as an asymptomatic thoracic dumbbell-shaped mass, incidentally discovered on a chest X-ray performed for unrelated reasons. MRI and CT demonstrated an intracanalar–endothoracic mass without bone erosion, radiologically indistinguishable from a benign nerve sheath tumor until histopathologic examination established the diagnosis.

#### 3.1.7. Histopathological and Immunohistochemical Findings

DTs typically originate from the proliferation of well-differentiated fibroblasts within fascial planes of soft tissues including muscle, aponeurosis, subcutaneous tissue, and neurovascular structures [51], infiltrating adjacent skeletal muscle cells. Histopathological examination, obtained either by surgery or needle biopsy, typically shows proliferated spindle cell fibroblasts and myofibroblasts embedded in a prominent collagenous stroma and vascular network, with occasionally accompanied by sarcolemmic giant cells [55]. Additional immunohistochemical and molecular pathological tests are crucial for diagnostic accuracy. Desmoid tumor cells usually exhibit strong immunoreactivity to β-catenin in the cytoplasm and nucleus [19,47]. This characteristic helps distinguish desmoid tumors from other tumors that show fibroblastic proliferation, such as solitary fibrous tumors or nodular fasciitis, which more commonly demonstrate positivity for CD34, vimentin, Bcl-2 and CD99 [10].

In our case, histopathology and immunohistochemistry, showing nuclear β-catenin positivity and negativity for CD34, S100 protein, and SMA, established the definitive diagnosis of desmoid fibromatosis. This underscores the diagnostic pitfalls and the critical role of tissue analysis in cases where imaging is inconclusive.

### 3.2. Surgical Management

Because of the rarity and the seemingly variable natural history of DTs, there is no specific method of treatment and no high-level evidence of its efficacy. The available recommendations are based on retrospective reviews and expert opinions [47]. Decision-making in DTs management is very complicated and must be planned based on a multidisciplinary approach [63].

Wide marginal resection is considered the major recommended approach. Although there are conflicting reports on the extent of margins to be excised, the wide marginal surgery should insure negative surgical margins [4,64]. However, spinal DTs often involve or abut critical neural and vascular structures, rendering radical excision technically challenging and potentially morbid. In such situations, a function-preserving approach, accepting marginal or subtotal resection, may be justified, particularly when combined with adjuvant therapies or close surveillance. This approach reflects a broader paradigm shift in the management of DTs: achieving long-term disease control while minimizing treatment-related morbidity.

In the literature, among 36 reported cases of spinal DTs, 4 patients underwent diagnostic preoperative needle biopsy: 2 of them have no reported data on treatment management nor follow-up, one patient underwent combined radio and chemotherapy, and one patient received radiotherapy alone. Thirty-two patients underwent surgery. Of these, 17 (53%) underwent wide resection, 8 (25%) marginal resection, and 7 (22%) subtotal/incisional resection (Table 2). Dumbbell-shaped lesions (6 cases reported in the literature, indicated by red asterisks in Table 1), extending both intra- and paraspinally, present specific anatomical challenges. Such tumors often require a tailored surgical strategy, sometimes involving combined anterior–posterior or costotransverse approaches to achieve adequate decompression while preserving spinal stability. Preoperative planning, including high-resolution imaging and interdisciplinary discussion, is therefore critical.

In this case, the procedure was planned in collaboration with the interventional radiologist, the thoracic surgeon, and the neurosurgical team. Preoperative angiography was performed to assess the arterial supply to the spinal cord. The surgical strategy included two phases: first, a left T10 laminectomy for removal of the intracanalar component, providing a view of the spinal cord and nerve root; second, removal of the intrathoracic component through a small thoracotomy between the left 10th and 11th ribs, allowing exposure of the intrathoracic mass (Figure 6).

### 3.3. Postoperative Adjuvant Therapy

Desmoid fibromatosis is a radio responsive tumor [63,65,66]: if tumor-free margins cannot be achieved (for cosmetic or functional reasons) postoperative radiotherapy should be considered as adjuvant therapy to improve local control [67]. Furthermore, for patients who lose the opportunity of surgery, radiotherapy can effectively control tumor progression and, on some occasions, can lead to complete or partial regression [68]. The total recommended dose of external radiotherapy is 50–60 Gy, with a dose of 1,8–2,0 Gy per exposure [69]. The radiotherapy should cover the whole region of the tumor, when possible, with the margin of radiotherapy about 5–8 cm away from the tumor [68]. Greenberg et al. reported an 89% rate of relapse-free survival for radiotherapy combined with surgery [70].

Other therapeutic options include hormonal drugs, anti-inflammatory therapy, and chemotherapy or a combination of all [46,71,72]. The higher incidence of DTs in women, mostly during pregnancy and postpartum, suggests that endocrine factors may be associated with DTs. It was therefore hypothesized that estrogen could modulate DT. Thus, drug treatment mainly consists of anti-estrogen therapy (tamoxifen). However, the evidence of the efficacy of this type of treatment has been limited to case series and single-arm trials [73].

Non-steroidal anti-inflammatory drugs (NSAIDs) may also have a role in treatment: sulindac, indomethacin and colchicine have been tested in patients with this disease, with varying degrees of success [74]. Overall, hormonal agents and NSAIDs have benign side effect profiles but generally limited efficacy. Cytotoxic chemotherapy, such as doxorubicin-dacarbazine or other chemotherapy regimens, may also be used [74]. Among patients with progressive, refractory or symptomatic DT, sorafenib, interferon-α and imatinib significantly prolonged progression-free survival [75].

This review showed that 6 patients received adjuvant therapy: 2 patients underwent postoperative radiotherapy, 3 patients received postoperative chemotherapy, and 1 patient underwent both adjuvant chemo and radiotherapy, showing complete tumor resolution, and no recurrence at follow-up.

In our case, the patient did not receive any adjuvant therapy.

### 3.4. Recurrence

In this review, we could collect follow-up data only in 24 patients, of which 23 were treated surgically, with a mean follow-up time of 26 months. Desmoid fibromatosis tends to infiltrate adjacent muscles microscopically, which cannot be determined in the operative field, yielding to a significant recurrence risk. In the literature, DT recurrence rates range from 24% to 77% depending on the patient’s age, location of the tumor, and resection margins [4]. Dahn et al. described recurrence rates of 70% with marginal excision and 8% with radical excision [49].

Of the 23 patients who underwent surgery and had follow-up, 7 (30%) experienced recurrence, with a mean time to recurrence of 7 months. A deeper analysis of recurrence rates, related to extent of resection, showed higher incidence in patients undergoing subtotal/incisional resection (50%) compared to those undergoing marginal (29%) or wide marginal resection (20%) (Table 2). However, due to limited data, these results were not statistically significant (*p* value 0.1–0.25).

### 3.5. Management of Recurrence

The recommended treatment of recurrent disease is repeated resection because it results in cure rates similar to those achieved with primary resection [55,65]. However, in a revision surgery it is very difficult to distinguish between scar tissue and tumor, which may result in inadequate safety margin for excision [66]. Revision surgery supplemented with radiation therapy results in better local control than surgery alone. Many authors reported local control rates between 78 and 81% for patients who received postoperative radiotherapy in comparison with 32–53% who were treated with surgery alone [68,70,76]. Among the 7 aforementioned cases with tumor recurrence, management was individualized based on tumor characteristics, prior treatment, and patient factors, resulting in cessation of tumor growth. Repeat surgery with margin resection was performed in 2 cases to achieve gross total resection while preserving neurological function [23,40]. Surgery with adjuvant therapy was performed in 3 cases to improve local control being complete resection limited by proximity to critical structures [6,10,43]. Definitive radiotherapy alone was utilized in 1 case, demonstrating that non-surgical approaches can provide effective local control [47]. One patient preferred observation of the suspected recurrence, that showed no further growth in follow-up MRIs [28].

In our case, all these therapeutic options were discussed with the patient as part of a shared decision-making framework. The patient declined further treatment recommendations. At 8-month follow-up, the residual/recurrent tumor remained stable in size over the subsequent 18 months. Ongoing radiological surveillance remains critical, as it allows timely intervention if the residual tumor shows signs of progression.

## 4. Conclusions

DTs are very rare tumors, especially those presenting as dumbbell-shaped neoplasms, with an endothoracic extension of this size.

Tumor etiology is evenly distributed between de novo and post-operatory origin, highlighting the contribution of surgical trauma in DTs pathogenesis: more studies are needed to understand this pathogenetic mechanism.

Combination of pathological results and the immunohistochemical finding of β-catenin in the cell cytoplasm and nucleus is essential for final diagnosis.

There is no standardized protocol for treating DTs. Wide marginal resection seems to be the major recommended approach. However, the extent of resection should take into account function preservation and minimize morbidity. Adjuvant treatments, including radio and chemotherapy, seem to improve local control. Lower recurrence rates have been reported for patients undergoing wide marginal resection (Table 2). Unfortunately, due to data paucity, these results are not statistically significant.

Given the lack of clear guidelines and of a significant number of cases reported so far, surgeons should refer to the best evidence available, the individual patient’s wishes, and the morbidity of the treatment. Close lifelong follow-up is paramount.

## Figures and Tables

**Table 1:** Clinical data from reported cases of spinal desmoid fibromatosis in the literature. Gray-highlighted rows indicate post-surgical cases. Red asterisks mark authors who reported cases presenting as spinal dumbbell tumors (six in total; note that Lacayo et al. reported four cases, but only one exhibited a dumbbell morphology).

Author and Year	Age	Gender	Presentation	Etiology	Period Between Surgery and Diagnosis of DT	Tumor Location	Treatment	Follow Up (Months)	Recurrence	Time to Recurrence	Recurrence Treatment
* Gonatas et al., 1961 [22]	45	F	painful mass	post-op	years	cervical paraspinal	marginal resection	14	no	/	/
Wyler et al., 1973 [23]	39	F	painful mass	post-op	12	cervicothoracic junction paraspinal	wide marginal resection	15	yes	8	wide marginal resection
* Friede et al., 1979 [24]	11	F	hypoesthesia, spasticity and paraparesis	de novo	/	T2–T5 intramedullary	intralesional resection	/	/	/	/
Oberthaler et al., 1988 [25]	21	M	painful mass	de novo	/	T10–L4 (bone)	intralesional resection + radiotherapy	36	no	/	/
Kriss et al., 1994 [26]	1.5	F	painless mass	de novo	/	cervical C2–C5 paraspinal	wide marginal resection	17	no	/	/
Ko et al., 1996 [27]	3	F	painless mass	de novo	/	T9–T10 intraspinal + mediastinal and chest wall	wide marginal resection	/	/	/	/
Maurer et al., 1996 [28]	18	F	painless mass	post-op	12	lumbar paraspinal	intralesional resection	6	yes	4	no
Lynch et al., 1999 [29]	49	F	painful mass	post-op	18	thoracic paraspinal	wide marginal resection	/	/	/	/
Shindle et al., 2002 [30]	12	F	pain, scoliosis and paraparesis	de novo	/	T7–T10 intraspinal, T9 and T10 root encasement, bone erosion + chest mass	Intralesional resection + radiotherapy	108	no	/	/
Guzey et al., 2006 [31]	50	F	painful mass	post-op	14	thoracolumbar junction paraspinal	wide marginal resection	14	no	/	/
Hara et al., 2007 [32]	50	M	shoulder pain	de novo	/	T3–T4 paraspinal	chemotherapy + radiotherapy	12	no	/	/
Cohen et al., 2008 [33]	27	F	painless mass	de novo	/	cervicothoracic paraspinal	chemotherapy + wide marginal resection	24	no	/	/
Chung et al., 2010 [34]	14	M	asymptomatic finding on routine imaging	Gardner syndrome	/	C7 vertebral body	marginal resection	12	no	/	/
Sonmez et al., 2011 [35]	55	F	pailful mass	post-op	6	thoracolumbar paraspinal	wide marginal resection	/	/	/	/
Sevak et al., 2012 [36]	48	F	painless mass	post-op	24	C7–T1 paraspinal	wide marginal resection	6	no	/	/
Shakur et al., 2013 [37]	45	F	neck and arm pain and paraesthesia	de novo	/	C5–T1 intraspinal- paraspinal	intralesional resection	40	no	/	/
	38	F	midthoracic back pain and paraesthesia	de novo	/	T9–T10 intraspinal-paraspinal	marginal resection + chemotherapy (tamoxifen)	10	no	/	/
Butel-Simoes et al., 2013 [38]	13	M	asymptomatic finding on routine imaging	Gardner syndrome	/	C7–T1 paraspinal	marginal resection + chemotherapy	48	no	/	/
Furlan et al., 2013 [39]	41	F	painful mass and low back pain	de novo	/	lumbar	/	/	/	/	/
Hood et al., 2013 [40]	25	F	painful mass	de novo	/	occipitocervical	subtotal resection + chemotherapy (imatinib)	27	yes	7	wide marginal resection
Kim et al., 2013 [41]	31	M	painful mass with sciatica	de novo		L3–L4 bone + paraspinal mass	marginal resection	/	/	/	/
Puvanesarajah et al., 2013 [42]	57	F	painful mass	post-op	10	thoracic paraspinal	wide marginal resection	24	no	/	/
* Eksi et al., 2015 [43]	63	F	low back pain, claudication and hypoesthesia	de novo	/	lumbar paraspinal	intralesional resection	24	yes	6	wide marginal resection + radiotherapy
Rispoli et al., 2015 [44]	57	F	painful mass	post-op	24	thoracic paraspinal	wide marginal resection	24	no	/	/
* Lacayo et al., 2016 [45]	48	M	painful mass	post-op	60	cervical paraspinal	radiotherapy	/	/	/	/
	53	M	painful mass	post-op	12	cervical paraspinal	wide marginal resection	/	/	/	/
	25	M	painful mass	post-op	48	cervical paraspinal	wide marginal resection	/	/	/	/
	61	M	painful mass	post-op	48	cervical paraspinal	/	/	/	/	/
Avincsal et al., 2018 [46]	71	M	painless mass	de novo	/	cervical paraspinal	wide marginal resection	84	yes	3	radiotherapy
* Goldstein et al., 2018 [47]	67	F	neck and bilateral shoulder pain	post-op	/	C2–C4 paraspinal	wide marginal resection	/	/	/	/
Bohl et al., 2019 [5]	56	F	painless mass	post-op	24	cervical paraspinal	wide marginal resection	1	no	/	/
Kumar et al., 2019 [6]	13	F	paraparesis	de novo	/	thoracic T2–T5 spinous process	marginal resection	48	yes	12	wide marginal resection + radiotherapy
* Luo et al., 2019 [7]	47	F	painless mass and bilateral cervico-brachialgia	de novo	/	cervical intraspinal-paraspinal	marginal resection + radiotherapy + chemotherapy	12	no	/	/
Mujtaba et al., 2020 [8]	42	F	neck pain and paraparesis	post-op	72	C7–T2 intraspinal-paraspinal	wide marginal resection	/	/	/	/
Schlag et al., 2022 [9]	58	M	painful mass	post-op	24	cervical paraspinal	wide marginal resection	12	no	/	/
Zheng et al., 2023 [10]	36	F	painless mass	post-op	11	cervical paraspinal	marginal resection	6	yes	6	wide marginal resection + radiotherapy

**Table 2:** Surgical extension of excision and recurrence rate.

Recurrence Rate	N. Patients with Recurrence	N. Patients with Follow-Up	N. Patients	Extent of Excision
20%	2	10	17	Wide
29%	2	7	8	Marginal
50%	3	6	7	Subtotal

## Data Availability

The original contributions presented in this study are included in the article. Further inquiries can be directed to the corresponding author.
